# Supplementary material for: Contrasted histories of organelle and nuclear genomes underlying physiological diversification in a grass species
Source: Proc Biol Sci. 2020 Nov 11;287(1938):20201960. doi: 10.1098/rspb.2020.1960 (PMC7735283; doi:10.1098/rspb.2020.1960)
Supplement: Supplementary Information [file rspb20201960supp1.pdf]

Supplementary material

Contrasted histories of organelle and nuclear genomes underlying physiological diversification in a grass species

Matheus E. Bianconi, Luke T. Dunning, Emma V. Curran, Oriane Hidalgo, Robyn F. Powell, Sahr Mian, Ilia J. Leitch, Marjorie R. Lundgren, Sophie Manzi, Maria S. Vorontsova, Guillaume Besnard, Colin P. Osborne, Jill K. Olofsson, Pascal-Antoine Christin

This Supplementary material contains six figures and two tables. Table S1 is in a separate .xlsx file.

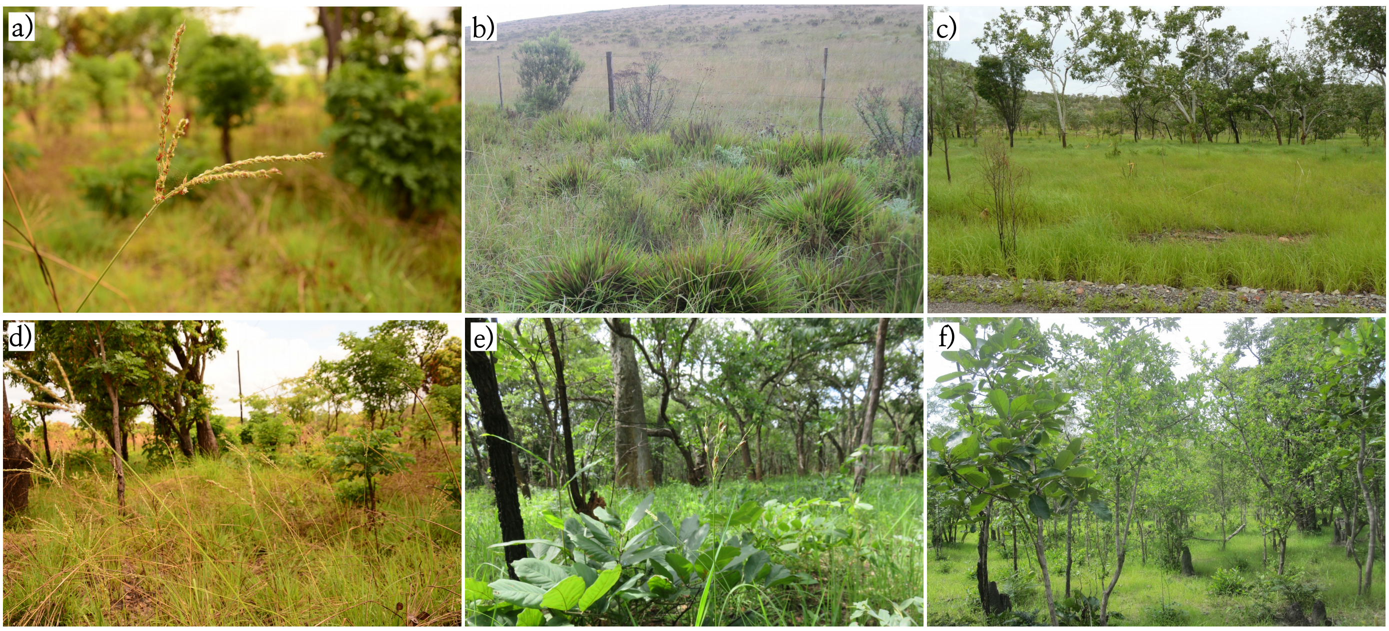

Fig. S1. Habitat diversity of *Alloteropsis semialata*. a) inflorescence of a C_4 hexaploid population in Mozambique (MOZ1601); b) tussocks of a C_3 diploid population in South Africa (RSA2); c) grassland in northern Australia where C_4 diploids can be found (population AUS4 in Olofsson *et al.* 2019); d) C_4 hexaploids in the lowland dry miombo woodlands of northern Mozambique (MOZ1601); e) C_4 hexaploids in the highland wet miombo woodlands of northwestern Zambia (ZAM1507); f) miombo woodlands in central Tanzania, where diploid C_3+C_4 populations can be found (TAN2).

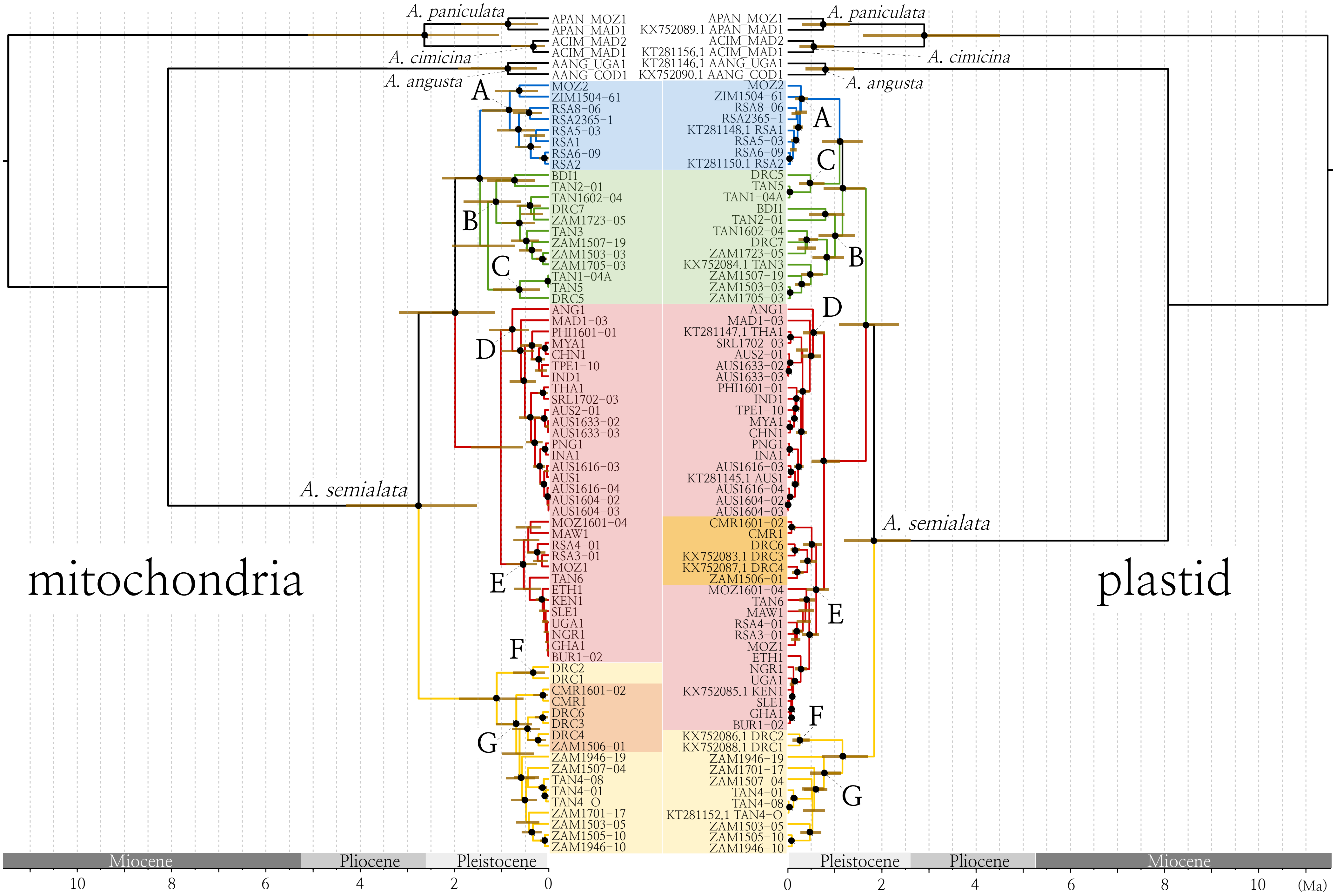

Fig. S2. Time-calibrated phylogeny of *Alloteropsis* based on mitochondrial (left) and plastid (right) genome sequences. Brown bars on nodes indicate 95% HPD. Circles highlight nodes with posterior probability ≥ 0.95. The organelle clades are indicated with letters (A-G).

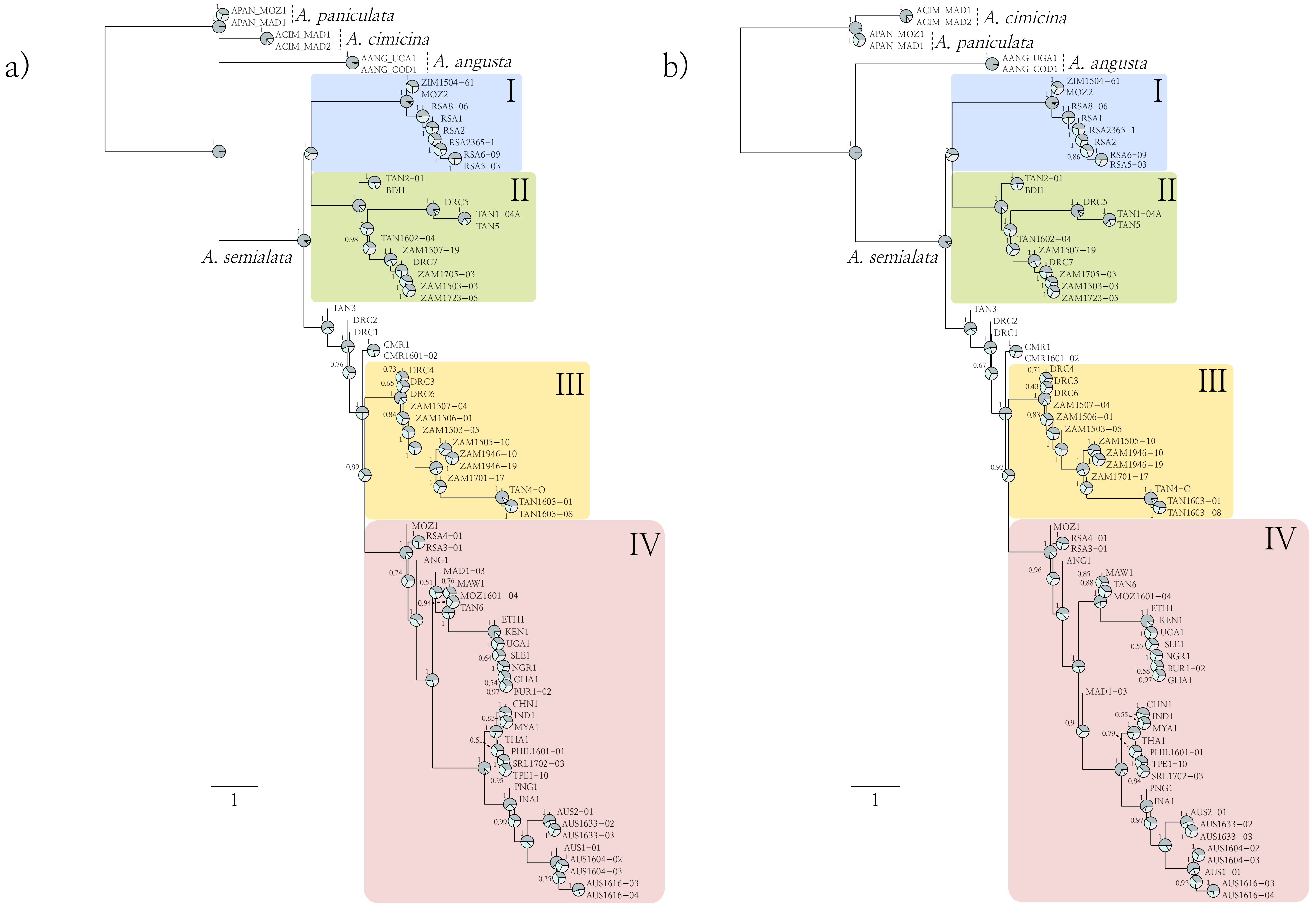

Fig. S3. Multigene coalescent species trees estimated from two datasets with less stringent filtering; a) alignments not trimmed (4,345 genes retained); b) sites with more than 70% of missing data were discarded (4,198 genes retained). Pie charts on nodes indicate the proportion of quartet trees that support the main (dark grey), first (pale blue) and second (light grey) alternative topologies. Local posterior probabilities are indicated near nodes. Branch lengths are in coalescent units.

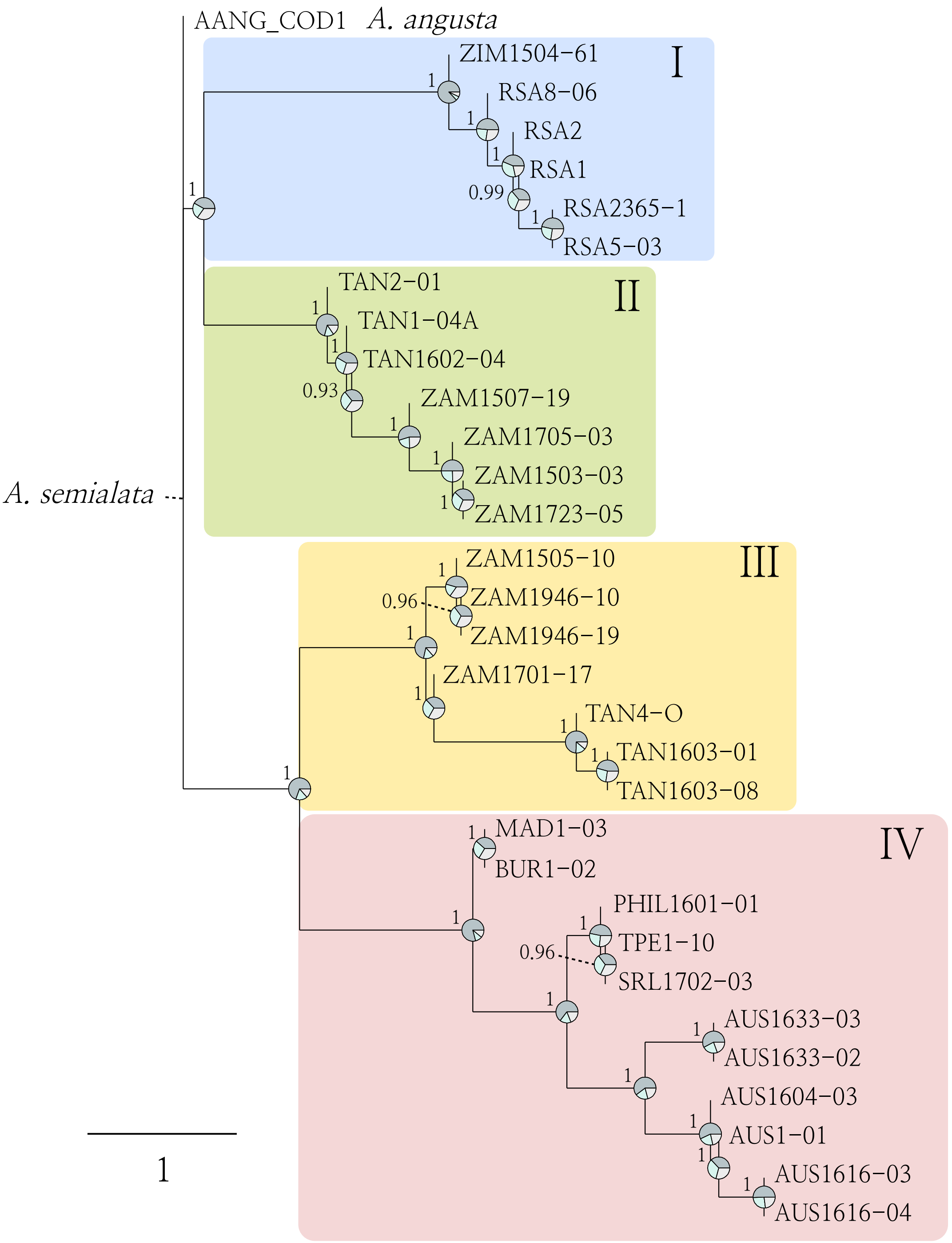

Fig. S4. Multigene coalescent species tree based on 3,553 genes. The gene set is as in Fig. 2, but only diploid individuals were retained. Pie charts on nodes indicate the proportion of quartet trees that support the main (dark grey), first (pale blue) and second (light grey) alternative topologies. Local posterior probabilities are indicated near nodes. Branch lengths are in coalescent units.

a)

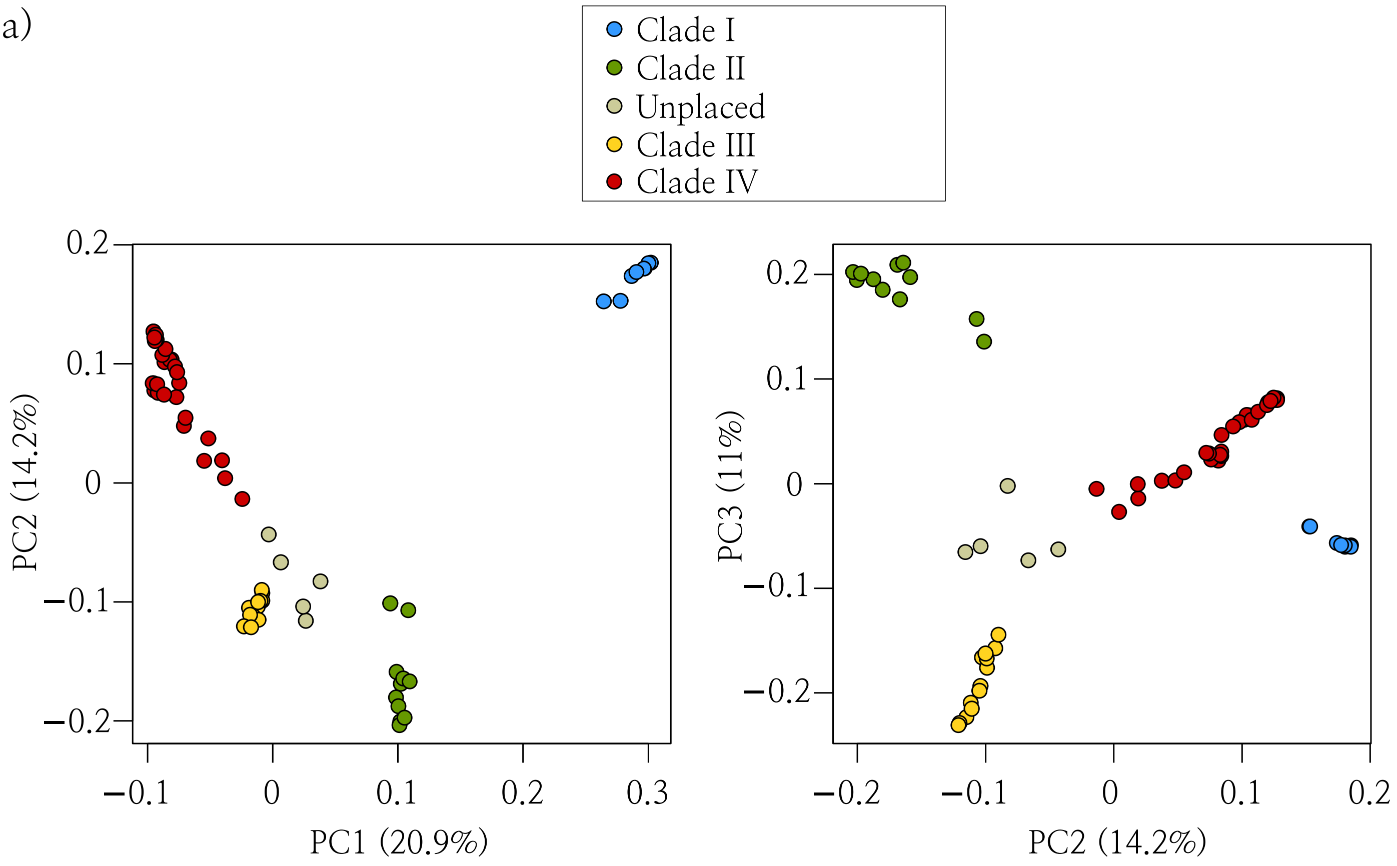

b)

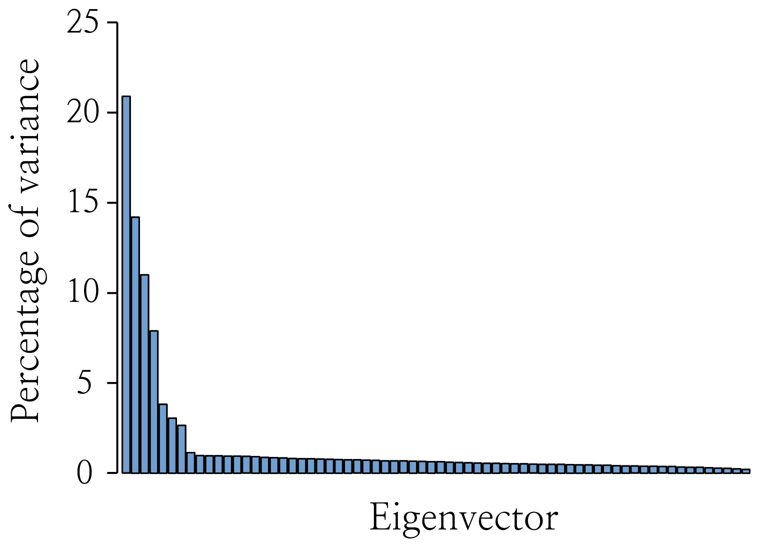

Fig. S5. Principal component analysis using genome-wide nuclear data of *Alloteropsis semialata.* a) The first and second axes (left), and the second and third axes (right). Colours correspond to major nuclear clades as identified in Fig. 2; b) ranked eigenvectors.

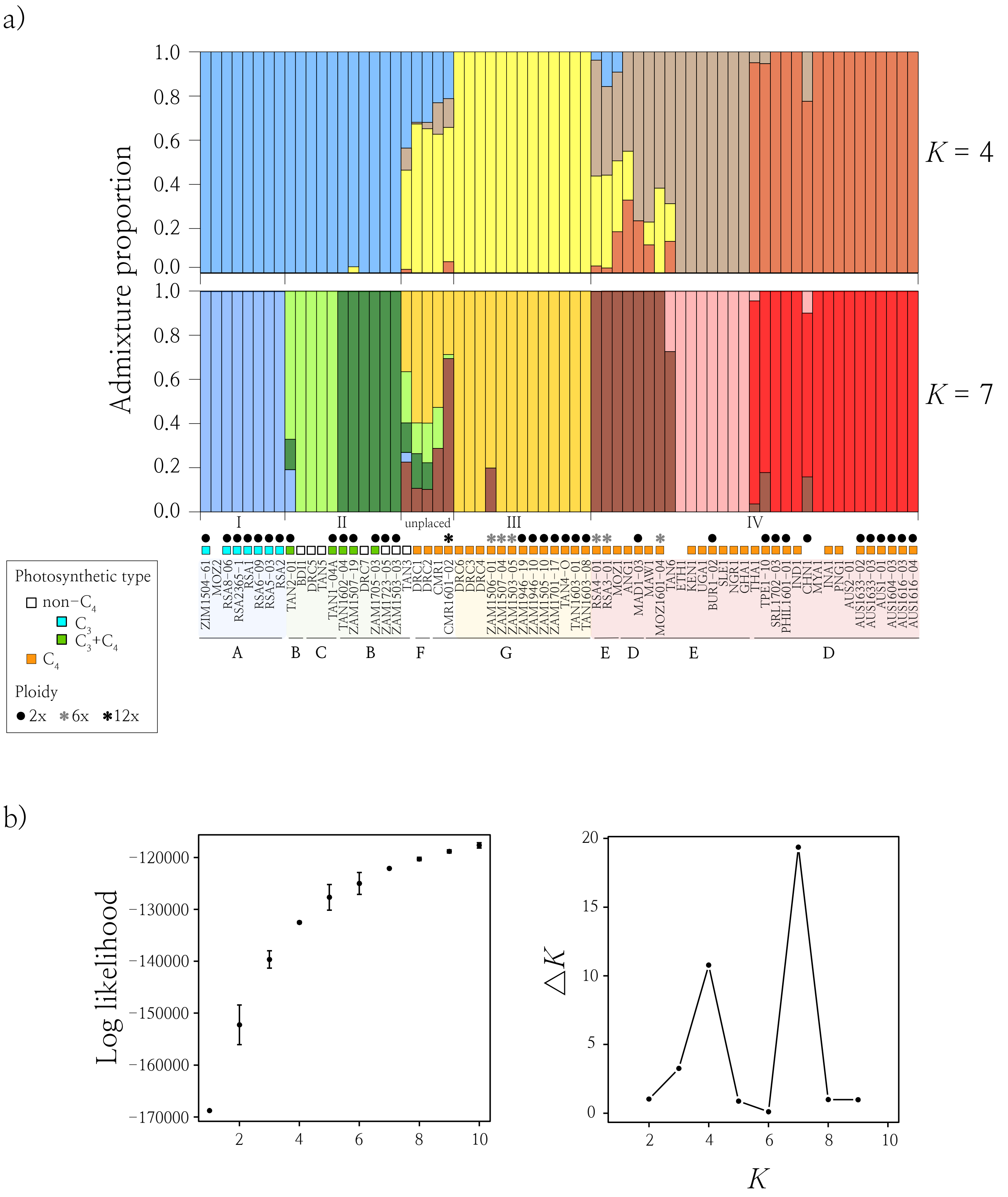

Fig. S6. Genetic structure of *Alloteropsis semialata*. a) Individual-based admixture analysis assigning individuals to the two best numbers of clusters (K). Nuclear clades (I-IV) and organelle lineages (A-G) are indicated at the bottom. b) (left) Mean likelihood (± SD) over five runs for each number of clusters tested for the admixture analysis (K:1-10); (right) fit improvement for the admixture analysis, as calculated according to Evanno *et al.* (2005).

Table S2. Genome composition analysis[a].

Accession	Nuclear clade	I	II	III	IV	Total[b]
RSA5-03	I	4750 (0.92)	273 (0.05)	77 (0.01)	37 (0.01)	5137
RSA8-06	I	675 (0.90)	60 (0.08)	10 (0.01)	6 (0.01)	751
RSA6-09	I	2233 (0.93)	119 (0.05)	24 (0.01)	14 (0.01)	2390
RSA2365-1	I	1552 (0.94)	65 (0.04)	20 (0.01)	19 (0.01)	1656
ZIM1504-61	I	1150 (0.78)	225 (0.15)	55 (0.04)	49 (0.03)	1479
TAN1-04B	II	346 (0.04)	8304 (0.86)	769 (0.08)	226 (0.02)	9645
TAN1602-04	II	47 (0.04)	942 (0.82)	99 (0.09)	62 (0.05)	1150
ZAM1503-03	II	41 (0.03)	1384 (0.92)	52 (0.03)	30 (0.02)	1507
ZAM1507-19	II	27 (0.03)	885 (0.93)	25 (0.03)	13 (0.01)	950
TAN2-01	II	144 (0.08)	1489 (0.82)	80 (0.04)	98 (0.05)	1811
TAN1-04A	II	52 (0.02)	2048 (0.93)	68 (0.03)	28 (0.01)	2196
ZAM1705-03	II	43 (0.04)	1096 (0.90)	62 (0.05)	19 (0.02)	1220
ZAM1723-05	II	67 (0.05)	1281 (0.90)	51 (0.04)	30 (0.02)	1429
ZAM1505-10	III	210 (0.03)	956 (0.11)	5710 (0.68)	1494 (0.18)	8370
ZAM1701-17	III	58 (0.03)	202 (0.10)	1481 (0.72)	323 (0.16)	2064
TAN1603-01	III	14 (0.01)	113 (0.07)	1480 (0.86)	122 (0.07)	1729
TAN1603-08	III	13 (0.01)	105 (0.07)	1336 (0.86)	104 (0.07)	1558
ZAM1506-01	III[c]	66 (0.04)	251 (0.14)	1168 (0.63)	355 (0.19)	1840
CMR1601-02	unplaced[d]	173 (0.07)	626 (0.27)	757 (0.33)	769 (0.33)	2325
RSA3-01	IV[c]	115 (0.09)	76 (0.06)	265 (0.21)	813 (0.64)	1269
RSA4-01	IV[c]	115 (0.11)	102 (0.10)	241 (0.23)	598 (0.57)	1056
MOZ1601-04	IV[c]	13 (0.02)	27 (0.04)	107 (0.15)	574 (0.80)	721
BUR1-02	IV	40 (0.03)	110 (0.08)	256 (0.18)	1014 (0.71)	1420
MAD1-03	IV	17 (0.02)	55 (0.06)	139 (0.15)	690 (0.77)	901
AUS1-01	IV	19 (0.00)	117 (0.02)	98 (0.02)	4531 (0.95)	4765
TPE1-10	IV	23 (0.00)	76 (0.01)	157 (0.02)	6505 (0.96)	6761
PHIL1601-01	IV	7 (0.01)	22 (0.02)	41 (0.03)	1235 (0.95)	1305
CHN1	IV	5 (0.01)	18 (0.02)	40 (0.04)	850 (0.93)	913
AUS2-01	IV	18 (0.01)	36 (0.02)	37 (0.02)	1548 (0.94)	1639
SRL1702-03	IV	15 (0.01)	33 (0.03)	49 (0.04)	1019 (0.91)	1116

[a] Phylogenetic assignment of alleles to each of the four major nuclear clades of *Alloteropsis semialata* (number of reads, with corresponding percentage between parentheses); [b] total number of reads assigned; [c] polyploid (6x); [d] polyploid (12x).

Supplementary references

Dunning LT, Lundgren MR, Moreno-Villena JJ, Namaganda M, Edwards EJ, Nosil P, Osborne CP, Christin P-A. 2017. Introgression and repeated co-option facilitated the recurrent emergence of C_4 photosynthesis among close relatives. *Evolution* **71**: 1541–1555.

Dunning LT, Moreno-Villena JJ, Lundgren MR, Dionora J, Salazar P, Adams C, Nyirenda F, Olofsson JK, Mapaura A, Grundy IM, *et al.* 2019a. Key changes in gene expression identified for different stages of C_4 evolution in *Alloteropsis semialata*. *Journal of Experimental Botany* **70**: 3255–3268.

Dunning LT, Olofsson JK, Parisod C, Choudhury RR, Moreno-Villena JJ, Yang Y, Dionora J, Paul Quick W, Park M, Bennetzen JL, *et al.* 2019b. Lateral transfers of large DNA fragments spread functional genes among grasses. *Proceedings of the National Academy of Sciences, USA* **116**: 4416–4425.

Evanno G, Regnaut S, Goudet J. 2005. Detecting the number of clusters of individuals using the software STRUCTURE: A simulation study. *Molecular Ecology* **14**: 2611–2620.

Lundgren MR, Besnard G, Ripley BS, Lehmann CER, Chatelet DS, Kynast RG, Namaganda M, Vorontsova MS, Hall RC, Elia J, *et al.* 2015. Photosynthetic innovation broadens the niche within a single species. *Ecology Letters* **18**: 1021–1029.

Lundgren MR, Christin P-A, Escobar EG, Ripley BS, Besnard G, Long CM, Hattersley PW, Ellis RP, Leegood RC, Osborne CP. 2016. Evolutionary implications of C_3–C_4 intermediates in the grass *Alloteropsis semialata*. *Plant, Cell & Environment* **39**: 1874–1885.

Lundgren MR, Dunning LT, Olofsson JK, Moreno-Villena JJ, Bouvier JW, Sage TL, Khoshravesh R, Sultmanis S, Stata M, Ripley BS, *et al.* 2019. C_4 anatomy can evolve via a single developmental change. *Ecology Letters* **22**: 302–312.

Olofsson JK, Bianconi M, Besnard G, Dunning LT, Lundgren MR, Holota H, Vorontsova MS, Hidalgo O, Leitch IJ, Nosil P, *et al.* 2016. Genome biogeography reveals the intraspecific spread of adaptive mutations for a complex trait. *Molecular Ecology* **25**: 6107–6123.

Olofsson JK, Dunning LT, Lundgren MR, Barton HJ, Thompson J, Cuff N, Ariyarathne M, Yakandawala D, Sotelo G, Zeng K, *et al.* 2019. Population-specific selection on standing variation generated by lateral gene transfers in a grass. *Current Biology* **29**: 3921–3927.
